# Quantifying Low Birth Weight, Preterm Birth and Small-for-Gestational-Age Effects of Malaria in Pregnancy: A Population Cohort Study

**DOI:** 10.1371/journal.pone.0100247

**Published:** 2014-07-01

**Authors:** Marcus J. Rijken, Alysha M. De Livera, Sue J. Lee, Machteld E. Boel, Suthatsana Rungwilailaekhiri, Jacher Wiladphaingern, Moo Kho Paw, Mupawjay Pimanpanarak, Sasithon Pukrittayakamee, Julie A. Simpson, François Nosten, Rose McGready

**Affiliations:** 1 Shoklo Malaria Research Unit, Mahidol-Oxford Tropical Medicine Research Unit, Faculty of Tropical Medicine, Mahidol University, Mae Sot, Thailand; 2 Centre for Molecular, Environmental, Genetic and Analytic Epidemiology, Melbourne School of Population and Global Health, The University of Melbourne, Melbourne, Australia; 3 Centre for Tropical Medicine, Nuffield Department of Medicine, University of Oxford, Oxford, United Kingdom; 4 Faculty of Tropical Medicine, Mahidol University, Bangkok, Thailand; Université Pierre et Marie Curie, France

## Abstract

**Background:**

The association between malaria during pregnancy and low birth weight (LBW) is well described. This manuscript aims to quantify the relative contribution of malaria to small-for-gestational-age (SGA) infants and preterm birth (PTB) in pregnancies accurately dated by ultrasound on the Thai-Myanmar border at the Shoklo Malaria Research Unit.

**Methods and Findings:**

From 2001 to 2010 in a population cohort of prospectively followed pregnancies, we analyzed all singleton newborns who were live born, normal, weighed in the first hour of life and with a gestational age (GA) between 28+0 and 41+6 weeks. Fractional polynomial regression was used to determine the mean birthweight and standard deviation as functions of GA. Risk differences and factors of LBW and SGA were studied across the range of GA for malaria and non-malaria pregnancies. From 10,264 newborns records, population centiles were created.

Women were screened for malaria by microscopy a median of 22 [range 1–38] times and it was detected and treated in 12.6% (1,292) of pregnancies. Malaria was associated with LBW, PTB, and SGA compared to those without malaria. Nearly two-thirds of PTB were classified as LBW (68% (539/789)), most of which 83% (447/539) were not SGA. After GA 39 weeks, 5% (298/5,966) of non-LBW births were identified as SGA. Low body mass index, primigravida, hypertension, smoking and female sex of the newborn were also significantly and independently associated with LBW and SGA consistent with previous publications.

**Conclusions:**

Treated malaria in pregnancy was associated with an increased risk for LBW, PTB, and SGA, of which the latter are most important for infant survival. Using LBW as an endpoint without adjusting for GA incorrectly estimated the effects of malaria in pregnancy. Ultrasound should be used for dating pregnancies and birth weights should be expressed as a function (or adjusted for GA) of GA in future malaria in pregnancy studies.

## Introduction

Pregnant women infected with *Plasmodium falciparum* and *P.vivax* malaria have an increased risk to deliver an infant with low birthweight (LBW; <2500 g) [1], which is associated with increased infant mortality [2]. Infants with LBW can be preterm births (PTB; <37 weeks gestation), small for gestational age (SGA; birthweight below the population specific 10^th^centile for the gestational age), or both [3]. These children have different short and long-term needs and outcomes [4]. Furthermore, SGA is associated with increased mortality in both preterm [5], [6] and term infants [7]. Despite the enormous burden of malaria in pregnancy in Africa [8], Asia-Pacific [9] and South America [8], progress on differentiating the relative contribution of PTB and SGA to LBW has been very slow.

To distinguish between PTB and SGA accurate pregnancy dating is required [10]. In resource-poor malaria endemic areas, dating has relied on gestational age (GA) estimations by the first day of the last menstrual period, symphysis fundal height or from newborn examinations using the Ballard or Dubowitz tests, which are all subject to wider intervals of accuracy than ultrasound dating [10]. However, accurate dating by early ultrasound (the current gold standard) is feasible in malarious areas [11], [12], highlighted in recently published trials [13]–[17].

When large numbers of pregnancies are dated precisely, and birth weights measured accurately [10], population-specific charts of mean birthweight and percentiles relative to GA can be developed. The objective of this study was to develop such charts from ultrasound dated pregnancies for a rural population on the Thai-Myanmar border. These GA-specific mean birthweight centiles were used to identify SGA newborns and more specifically to quantify the relative contribution of malaria infections in pregnancy to the proportion of LBW, PTB, and SGA. A recent analysis in low and middle income countries including 20 cohorts and 2,015,019 live births from Asia, Africa, and Latin America confirmed the low proportion but high mortality risk of PTB, and the increased risk of early and late neonatal mortality with SGA [4].

## Methods

### Ethics statement

This is a retrospective hospital record analysis. For those patients in trials a written informed consent was obtained including storing of data and samples. For the women seen in the ANC the routine clinical records were anonymized and these pregnancy records have been routinely entered into a database since 1987. Permission was granted by Oxford Tropical Research Ethics Committee (reference: OXTREC 28–09) to use these records for analysis.

### Study site and population

The Shoklo Malaria Research Unit (SMRU) is located on the border between Thailand and Myanmar, a malaria endemic area where *P.falciparum* and *P.vivax* transmission is low and seasonal. Since the inception of the antenatal care program in 1986, all pregnant women have been encouraged to attend in the first trimester and return for weekly malaria screening and treatment and routine obstetric care. In 2001 antenatal ultrasound was introduced to improve GA estimation in this population with low literacy rates [18] and difficulties with last menstrual period dating due to the use of different calendars or absence of calendar [19]. Locally trained health workers (10 sonographers) obtained all ultrasound scans using Toshiba Powervision 7000 (since 2006), Dynamic Imaging (since 2001), and Fukuda Denshi UF 4100 (since 2002) ultrasound scanners. Their practice was supervised by doctors certified in fetal ultrasound. At the booking visit, ultrasound was used to determine viability, identify multiple pregnancies and estimate GA by Crown Rump Length (CRL) before 14 weeks GA; and fetal head (biparietal diameter (BPD) or head circumference (HC)) measurement between 14 and 24 weeks GA as recommended by the British Medical Ultrasound Society at the time. Each biometry measurement was routinely obtained twice, as part of the quality control system [19]. The training manual and protocol for obtaining trans-abdominal CRL and biometry measurements was based on recommendations from the British Medical Ultrasound Society [20]. Measurements after 24 weeks GA are unreliable for accurate pregnancy dating [21], [22].

### Outcomes and SMRU delivery rooms

All women were encouraged to deliver in the SMRU facilities under supervision of trained skilled birth attendants and midwives from the same population. Each labor was monitored using a WHO partogram. All medical and obstetric problems were investigated and treated by locally trained health workers in SMRU facilities and supported by doctors. Women requiring Caesarean section were referred to the nearest Thai hospital. Each baby was weighed within 1 hour on an electronic SECA (Model 336 or 376, accuracy = 10 g) digital newborn scale. All infants born in SMRU delivery rooms have a standardized newborn surface examination done by formally trained staff. Abnormalities that were obvious by ultrasound (the sonographers had no official training for anomalies) were verified by an obstetric doctor and, when conformed, were excluded from analysis.

### Diagnosis of malaria

Malaria was diagnosed by Giemsa stained thick and thin blood films; 200 fields on the thick film were read before being declared negative. Malaria infection was defined by the presence of asexual stages of Plasmodium (*P.vivax* or *P. falciparum*) in the peripheral blood irrespective of maternal symptoms. There was no presumptive treatment of malaria or chemoprophylaxis. Women were treated according to the WHO malaria guidelines. In this analysis, women were counted as malaria positive when they had at least one documented malaria infection in pregnancy.

### Definitions

GA was expressed in weeks (X) and number of days (+y): X^+y^. GA was calculated from the mean of two first trimester CRL measurements at the first antenatal visit (before GA 13^+6^ weeks) using an established formula [23] or the mean of two BPD measurements (before 23^+6^ weeks) using an Asian formula [24]. Hypertension was defined as having a diastolic blood pressure > = 90 mm Hg and/or a systolic blood pressure > = 140 mm Hg with or without proteinuria. Miscarriage was defined as a pregnancy ending before 28 weeks GA and stillbirth as a delivery from 28 weeks or ≥800 g birthweight in which the infant displayed no sign of life (gasping, muscular activity, cardiac activity). The 28-week GA, rather than the current WHO 22-week GA cut-off was chosen, as no infant ventilatory support was available in the clinics. This cut-off has been in place as the lower limit of viability since SMRU was established in this area. Congenital abnormality was defined as any major abnormality identified during newborn examination after birth. Low birthweight was defined as a birthweight less than 2500 grams. Preterm births was defined as birth before 37 weeks gestation), small for gestational age as a birthweight below the population specific 10^th^centile for the gestational age (or birth weight z-score less than 1.28 for a given GA using a modeled BW/relationship as described below).

### Inclusion and Exclusion criteria

The cohort included women who attended the SMRU ANC between 2001 and 2010, had a CRL or BPD measurement before 24 weeks, delivered a live-born, congenitally normal singleton infant at SMRU delivery rooms, with a gestation within 28^+0^ to 41^+6^ weeks, and whose newborn was weighed within the first hour after birth.

### Statistical Analysis

Clinical data and the results of the ultrasound scans were entered into a Microsoft Access database. Using R software version 2·15·2, fractional polynomial regression analysis [25], [26] was used to model mean birthweight and standard deviation as functions of GA. This allows for GA-varying attributes of both the mean and standard deviation of birthweight to be captured in the fitted models. In order to select the best fitting model, the model selection procedure described by Ambler and Royston [27] was followed using the ‘mfp’ package in R [28]. To assess the goodness of fit of the fitted models, the z-scores (calculated for each GA) were computed using the equation:
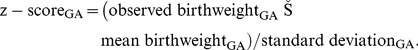
(1)


The z-scores obtained this way should be approximately normally distributed and this was assessed by visual inspection of normal probability plots. As these z-scores have been adjusted for GA, a plot of z-scores versus gestational age should be randomly scattered around zero with approximately 95% of the values lying between the limits ±1·96.

Centile curves were obtained using the equation:
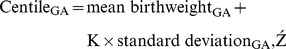
(2)where K is the corresponding centile of the standard normal distribution. For example let K = ±1·96 for the 2·5th and 97·5th centiles to obtain the 95% reference interval. Observations which fell beyond a specified reference interval (equivalent to a specified z-score) can be regarded as unusual or extreme. Using these reference curves, SGA births were identified in the current population and these data were subsequently analyzed. The association of birthweight and SGA births with malaria, primigravida status, hypertension, smoking, age, body mass index (BMI) of the mother and the sex of the baby was explored using univariable and multivariable linear regression models, with adjustment for GA. Effect modification of the association between birthweight and GA by malaria was also assessed by including an interaction term. When the likelihood ratio test was used to compare this with a model with no interaction term, no effect modification was observed (p-value = 0·86). Proportions of LBW, PTB, and SGA in malaria and non-malaria pregnancies were compared using chi-squared tests. The manuscript is written according to the STROBE statement guidelines.

## Results

**Figure 1:**
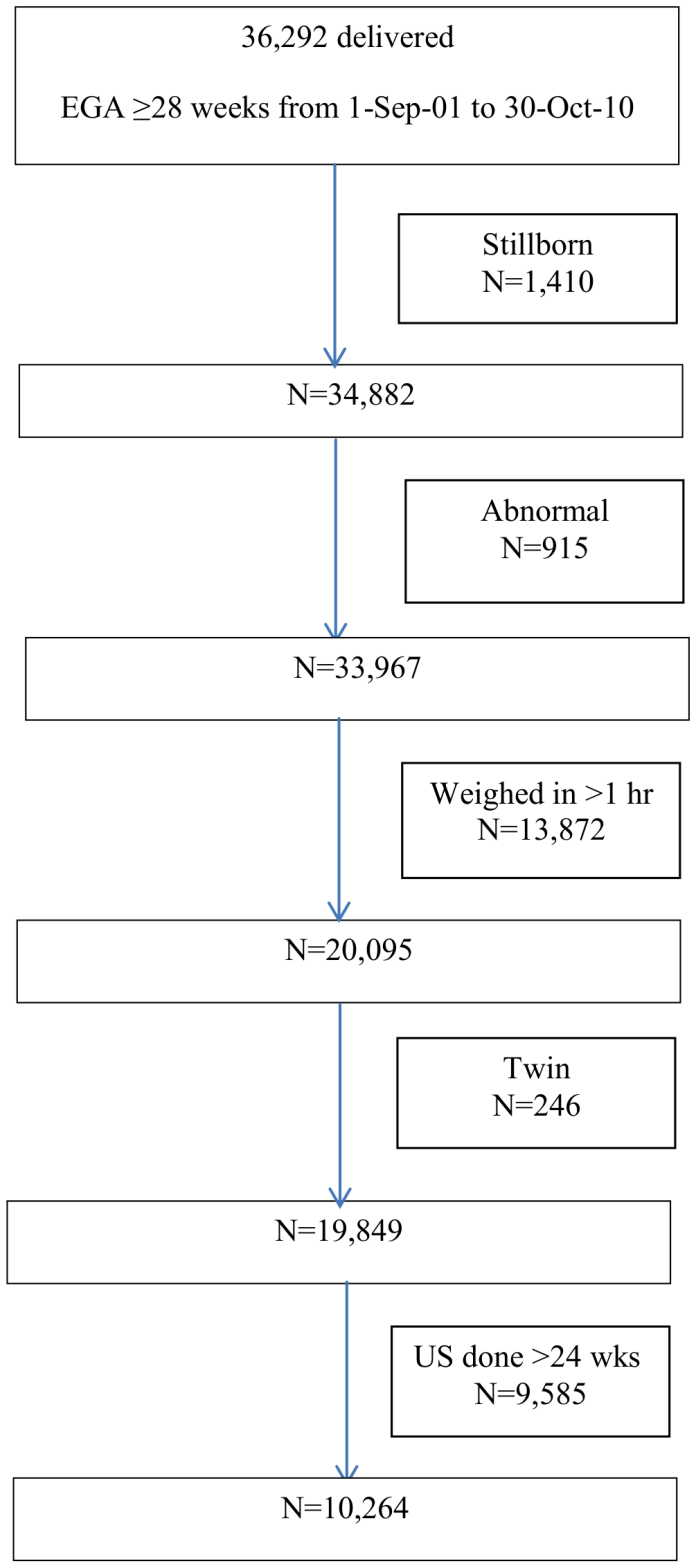
Study Population Inclusion.

10,264 pregnant women attended the SMRU ANC between 2001 and 2010 and met the inclusion criteria (Figure 1). They had a mean age of 26 (SD 7; range 14–48) years and 27·1% (2,778/10,264) were primigravida. The mean GA at delivery was 38^+6^ weeks (SD 1^+1^; range 28^+2^ to 41^+6^), and 51·4% (5276/10,264) of the babies were male. Women were screened for malaria by microscopy a median of 22 (IQR 16–28) [range 1–38] times. Malaria infection was detected in 12·6% (1,292/10,264) of pregnancies. Overall, 7·7% (789/10,264) of women in this birth cohort had a PTB. The overall mean birthweight was 2953 (SD 454) g; 2906 (SD 434) g for girls and 2998 (SD 468) g for boys.

### Birthweight/GA population chart

In 65% (6672/10264) of the women the GA was determined by CRL and in the remaining 35% (3592/10,264) of the cohort by BPD. The percentage of women with malaria was similar in these groups (percentage difference = 0·3; p-value = 0·6). The difference in the mean GA at delivery between the two groups was small (273 days in the CRL group and 274 days in the BPD group) so all women were included in the analysis. The best fitting curves for the birthweight mean and standard deviation were found to be a two-term fractional polynomial with powers (3;3) and a one-term fractional polynomial with power 1 (equivalent to a simple linear regression) respectively. The equations to obtain the fitted values (GA in days) were as follows:
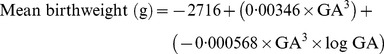
(3)

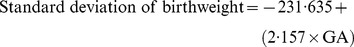
(4)


**Figure 2:**
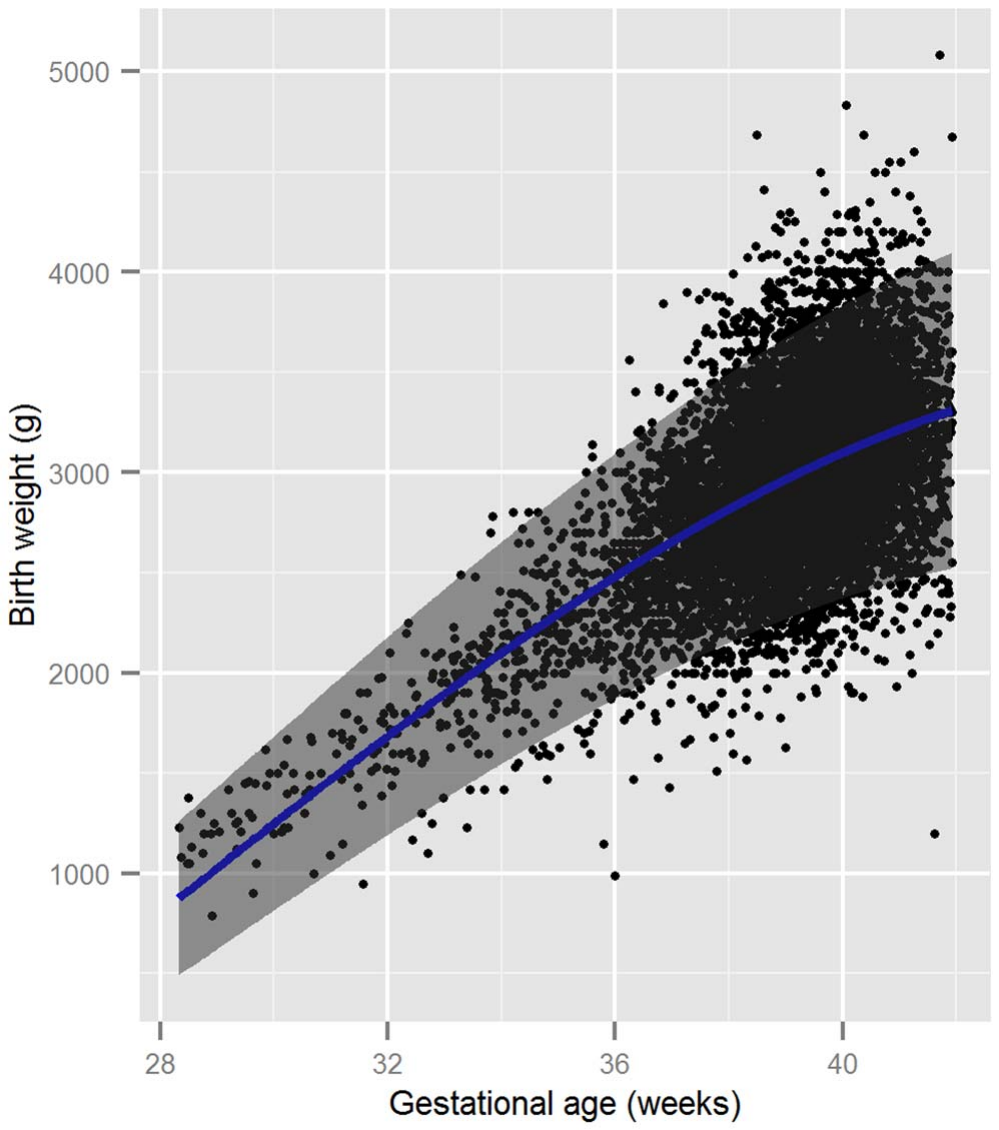
Raw data (N = 10,264) showing the fitted mean birthweight versus gestational age curve along with 2·5^th^ and 97·5^th^centiles.

The fitted values for mean birthweight and standard deviation were substituted into equation (2) to obtain the centile curves (Figure 2). The mean birthweight and reference intervals are provided in Table S1.

For a new observation within the range of the GA of this population, the expected mean birthweight and standard deviation can be calculated using equations (3) and (4) respectively. Equation (1) can then be used to obtain the z-score. A z-score below −1·28 indicates a SGA birth. Using the standard normal distribution, one can then obtain the corresponding centile. For instance according to the fitted models a baby with low birthweight (2500 g) at week 34 has a z-score of 1·423 corresponding to 92·2^th^ centile so is not SGA, whereas the same birthweight (2500 g) at 41 weeks gestation has a z-score of −1·846 lying on the 3·2^th^ centile and therefore is classified as SGA. The importance of accurate estimation of GA is highlighted by the following example. A baby born at week 35 weighing 2100 gram is premature but not SGA. In contrast a baby born with the same weight (2100 g) at week 38 is not premature but is SGA.

We used these fitted models to identify SGA births in the current population. The proportions of LBW, PTB, and SGA were significantly higher for pregnancies with malaria compared to those without malaria, although the risk difference for PTB was low (Table 1). The risk of LBW was 17·4% (225/1292) and 11·9% (1072/8972) in malaria and non-malaria pregnancies respectively (P<0.01).

**Table 1:** Newborn characteristics classified by malaria infection in pregnancy on the Thai-Myanmar border 2001–2010 (n = 10264).

	Non Malaria	Malaria	Risk difference (95% Confidence Interval)	P value
	n = 8972	n = 1292		
Small for gestational age, % (n)	9.1 (820)	13.9 (179)	4.8 (2.7, 6.7)	<0.01
Small for gestational age from the global reference curve, % (n) [34]	21.2 (1906)	28.9 (374)	7.7 (5.0,10.4)	<0.01
Low birth weight, % (n)	11.9 (1072)	17.4 (225)	5.5 (3.2, 7.7)	<0.01
Preterm birth, % (n)	7.5 (670)	9.2 (119)	1·7 (0.03, 3.5)	0.03

n = number.

**Figure 3:**
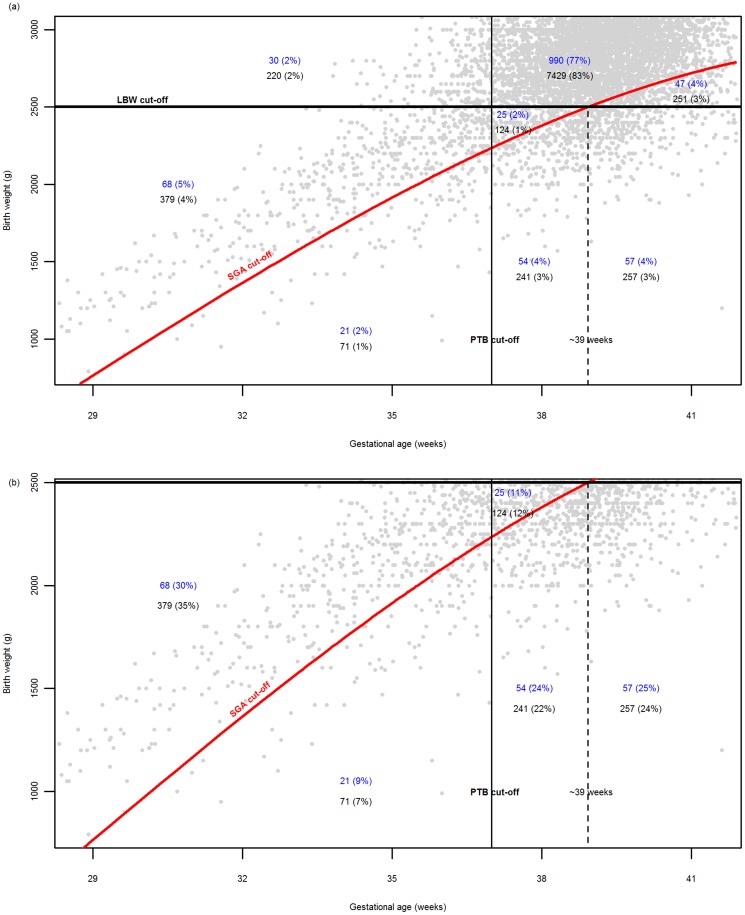
Plots showing the relationship between LBW, PTB, and SGA, illustrating the proportions of births for each category in (a) the whole population (b) only among LBW births. The numbers (%) indicate the malaria (blue) and non-malaria (black) pregnancies. Grey points represent births, and the percentages are rounded off to the nearest whole number.

Figures 3 (a) and (b) provide a graphical illustration of the relationship between LBW, PTB, and SGA. PTB contributed to 39% (89/225) of LBW in malaria pregnancies and 42% (450/1072) of LBW in non-malaria pregnancies (Figure 3(b)). A large proportion of babies classified as LBW were PTB but not SGA: 30% (68/225) and 35% (379/1072) in malaria and non-malaria pregnancies respectively (Figure 3(b)). These 447 births would have been categorized as “double” adverse outcomes (LBW and PTB) while in fact their weights were adequate for their GA. In contrast between 39 and 41 weeks GA in which over 60% (6,280/10,264) of women delivered, more infants were SGA than were LBW (Figure (3(a)).

The percentages of SGA and LBW by malaria infection in pregnancy for each week of GA are summarized in Table 2. As expected before 34 weeks virtually all newborns were LBW but the number of malaria cases available for analysis was very small. From 34 weeks GA onwards, the proportion of both LBW and SGA was increased in those infected by malaria compared to non-malaria pregnancies. From 34 weeks gestation onwards the overall proportion of SGA newborns for each GA week varied from 10·1–23·3% and was higher in malaria infected pregnancies compared to 8·0–13·7% in non-malaria infected pregnancies but these proportions were not adjusted for other factors associated with SGA.

**Table 2:** Percentage of small-for-gestational-age and low birthweight newborns in non-malaria and malaria infected pregnancies for each week of gestational age.

Gestational age	Non- Malaria	Malaria	SGA	SGA	LBW	LBW
Week	n	n	n (%)	n (%)	n (%)	n (%)
			Non-Malaria	Malaria	Non-Malaria	Malaria
28	8	3	0 (0·0)	0 (0·0)	8 (100·0)	3 (100·0)
29	14	5	0 (0·0)	0 (0·0)	14 (100·0)	5 (100·0)
30	16	4	1 (6·2)	0 (0·0)	16 (100·0)	4 (100·0)
31	24	5	3 (12·5)	0 (0·0)	24 (100·0)	5 (100·0)
32	33	8	2 (6·1)	2 (25·0)	33 (100·0)	8 (100·0)
33	61	12	5 (8·2)	1 (8·3)	59 (96·7)	12 (100·0)
34	97	10	13 (13·4)	2 (20·0)	83 (85·6)	9 (90·0)
35	117	29	10 (8·6)	6 (20·7)	77 (65·8)	24 (82·8)
36	300	43	37 (12·3)	10 (23·3)	136 (45·3)	19 (44·2)
37	804	125	76 (9·4)	21 (16·8)	164 (20·4)	38 (30·4)
38	1993	273	165 (8·3)	33 (12·1)	201 (10·1)	41 (15·0)
39	2896	383	263 (9·1)	52 (13·6)	172 (5·9)	38 (9·9)
40	1965	298	157 (8·0)	30 (10·1)	60 (3·0)	9 (3·0)
41	644	94	88 (13·7)	22 (23·4)	25 (3·9)	10 (10·6)

SGA: small-for-gestational-age, LBW: low birthweight, n: number.

Regression models were used to explore the association of birthweight and SGA births with malaria, primigravida status, hypertension, smoking, age, body mass index (BMI) of the mother and the sex of the baby. Malaria during pregnancy, primigravida, hypertension, smoking and female sex of the newborn were found to be associated with lower birthweight (Table 3) and SGA (Table 4) while the age of the mother was not statistically significantly associated with birthweight or SGA. Each additional full point in BMI was associated with an increase in birthweight of 24 grams, whereas malaria infection in pregnancy was associated with a reduction in birthweight of 50 grams (Table 3).

**Table 3:** Risk factors associated with birth weight (grams) at the Thai-Myanmar border, 2001–2010, using multivariable linear regression with adjustment for gestational age#.

			Mean weight difference (in grams)^a^		Mean weight difference (in grams)^b^	
		Frequency (%)	Coefficient (95% CI)	P value	Coefficient (95% CI)	P value
BMI	kg/m^2^	8639	26·93 (24·36, 29·51)	<0·01	23·64 (21·06,26·21)	<0·01
Maternal Age	years	10264	3·32 (2·25, 4·40)	<0·01	−0·91 (−2·23,0·42)	0·20
Malaria	No	8972 (87·4)				
	Yes	1292 (12·6)	−80·79 (−102·20,−59·39)	<0·01	−50·33 (−72·2, −28·50)	<0·01
Hypertension	No	9929 (96·7)				
	Yes	335 (3·3)	−69·36 (−109·43,−29·29)	<0·01	−101·08 (−141·58,−60·59)	<0·01
Smoking	No	7893 (76·9)				
	Yes	2316 (22·6)	−93·12 (−110·07,−76·18)	<0·01	−106·40 (−125·44,−87·37)	<0·01
Sex of baby	M	5276 (51·4)				
	F	4988 (48·6)	−122·54 (−136·61,−108·48)	<0·01	−120·65 (−135·34,−105·96)	<0·01
Primigravida	No	7486 (72·9)				
	Yes	2778 (27·1)	−126·68 (−142·58,−110·79)	<0·01	−130·38 (−149·35,−111·41)	<0·01

# To account for the non-linear association between birthweight and gestational age, gestational age was included in these models as the non-linear terms given in equation (3).

a adjusted for gestational age only.

b adjusted for gestational age and the other risk factors in the table.

**Table 4:** Risk factors associated with SGA at the Thai-Myanmar border, 2001–2010, using univariable and multivariable logistic regression.

		Univariable logistic regression		Multivariable logistic regression	
		Odds Ratio (95% CI)	P value	Odds Ratio (95% CI)	P value
BMI	kg/m^2^	0·86 (0·84,0·89)	<0·01	0·87 (0·84, 0·89)	<0·01
Maternal Age	years	1·00 (1·00, 1·02)	0·14	1·00 (1·00, 1·02)	0·14
Malaria	No				
	Yes	1·60 (1·35,1·90)	<0·01	1·35 (1·12,1·64)	<0·01
Hypertension	No				
	Yes	2·00 (1·50, 2·68)	<0·01	2·13 (1·54, 2·95)	<0·01
Smoking	No				
	Yes	2·00 (1·74, 2·30)	<0·01	2·16 (1·82, 2·57)	<0·01
Sex of baby	Male				
	Female	1·90 (1·66, 2·18)	<0·01	1·94 (1·67, 2·25)	<0·01
Primigravida	No				
	Yes	1·62 (1·41, 1·86)			
	<0·01	2·26 (1·87, 2·72)	<0·01		

## Discussion

In this large cohort of pregnant women living in a malarious area, GA was obtained by ultrasound and malaria episodes were detected and treated by frequent screening. As expected malaria infection was a significant risk factor for LBW but also for SGA and to a much lesser extent for PTB (Table 1). Other known factors associated with LBW and SGA such as primigravida, hypertension and smoking were also observed in this population indicating the robustness of the data [3]. This is the largest single study able to differentiate the respective contribution of malaria infection in pregnancy on LBW, PTB, and SGA and not biased by late assessment of GA, inclusion of stillbirths, multiple pregnancies, congenitally abnormal infants, or infants weighed more than 1 hour after birth [10].

Infants born preterm and SGA rather than only LBW have the highest risk of mortality in low-income and middle income countries [4], [6]. Most PTB were LBW (68% (539/789)) but not SGA 83% (447/539). In the malaria group 25% (30/119) were PTB but not LBW (Table 2). By contrast after 39 weeks GA there were 5% (298/5966) of births not classified as LBW that were SGA in particular in the malaria group (Figure 3 (a) and Table 2). Hence studies that use LBW not adjusted for GA as an outcome of pregnancy [29] may misrepresent the impact of malaria: over-estimating for PTB and under-estimating for term births. Nevertheless LBW remains one of the most frequent primary outcomes measured in malaria in pregnancy studies due to the shortage of well dated pregnancies and because it is convenient and easy to measure. The combination of ultrasound dating, precise methods of birthweight measurements and population centiles are required to correctly quantify SGA and PTB and the subsequent increased risk for mortality in malaria affected pregnancies [10].

The marginally higher risk difference (Table 1; risk difference 1.7% units, P = 0.03), observed for PTB in those women exposed to malaria during pregnancy was small and this effect may not be clinically important. A recent study from the same population suggests that changes in growth can be observed early in gestation (before 24 weeks) [14], highlighting the need to provide adequate prevention of any infection by malaria parasites even in the first trimester [9], [30]. While ultrasound remains the best method of GA assessment, an infection that causes growth restriction before the dating scan could result in an over-estimate of PTB. In a large dataset this can potentially cause a small but statistically significant left shift in the GA data for the group with infection.

A limitation of using SGA is that it still encompasses neonates that are constitutionally small and those that are pathologically small due to fetal growth restriction [3], [31]. The diagnosis of fetal growth restriction due to malaria can only be made by demonstrating significant deviation from the normal pattern of intra uterine growth. This requires serial and regular ultrasound measurements and frequent malaria screening as the timing of malaria infection cannot be controlled for [32]. Such studies are currently being conducted in an attempt to elucidate this question (www.interbio21.org.uk/) [17], [33].

This prospective observational cohort study has some limitations. Due to the long study period subsequent pregnancies of a woman may have been included in the database. While the birth outcomes of a single woman (e.g. repeated premature labour not related to malaria) may have biased the results the sample size of this cohort is large and the bias small. Despite the overall size of the cohort the number of severely premature infants remained small and significant effects of malaria on the length of gestation prior to 34 weeks were not observed. More detailed analysis and modeling of data to describe the relative contribution to SGA from the two dominant malaria species (*P.falciparum* and *P.vivax*), GA at time of infection, symptoms and severity at the time of treatment and repeated episodes of malaria infections is in preparation. In this analysis most of the recommendations for birthweight analysis [10] were met but head circumference and length of the newborn were not systematically recorded in all newborns. In the 35% of infants dated from 14 to 24 weeks only head measurements were used to estimate GA in alignment with recommendations at the time of introduction of ultrasound to the setting. However, a combination of multiple biometric parameters (biparietal diameter, head circumference, abdominal circumference, and/or femur length) may be recommended to determine GA, rather than a single parameter [11],[21].

A population specific chart of mean birthweight and centiles was created for the rural population on the Thai-Myanmar border and is clinically relevant for ongoing obstetric and midwifery care based on data collected over 10 years. As many resource poor settings lack the infrastructure to accomplish such a large sample size the idea of a global reference for birthweight percentiles based on 100 well dated pregnancy outcomes is appealing [34]. However in this setting (approximately 2500 deliveries per year) it took 523 deliveries and 15 months from the first scan to the last delivery to reach the first 100 such pregnancy outcomes implying that simple suggestions surrounding ultrasound dating still require a significant investment in resources, manpower, training and ongoing support particularly for quality control [11], [12]. When the global reference for birthweight percentiles was applied to this data [34], 1281 (12.5%) extra pregnancies were classified as SGA, compared to the number of SGA pregnancies obtained using the local population centiles, indicating the significant burden of this problem in these vulnerable populations. However, in this population, the centiles presented in this manuscript may be the most appropriate for the identification of fetuses that are SGA.

In conclusion, this study demonstrates that despite active detection and treatment, malaria in pregnancy is associated with an increased risk for SGA in this population. The proportion of SGA varies with GA and affects a higher proportion of infants than LBW in the majority of infants born from 39–41 weeks of gestation. In areas with less active detection and late treatment of malaria in pregnancy the effects of malaria may be even more pronounced. LBW as a proxy for SGA may inadvertently miscalculate the impact of malaria in pregnancy. Tools that promote health worker recognition of SGA rather than LBW in resource constrained settings may allow more targeted management for at risk neonates and help reduce child mortality [4]. Early ultrasound before 24 weeks and preferably before 14 weeks, should be used for dating pregnancies and birth weights should be expressed by the GA in future malaria in pregnancy studies. Safe and effective methods to prevent malaria in pregnancy remain a priority.

## Supporting Information

Table S1 Birthweight (g) percentiles per completed gestational week for the refugee and migrant population on the Thai-Myanmar border.
(DOCX)
