# Supplementary material for: Quantifying Low Birth Weight, Preterm Birth and Small-for-Gestational-Age Effects of Malaria in Pregnancy: A Population Cohort Study
Source: PLoS One. 2014 Jul 1;9(7):e100247. doi: 10.1371/journal.pone.0100247 (PMC4077658; doi:10.1371/journal.pone.0100247)
Supplement: Table S1 — Birthweight (g) percentiles per completed gestational week for the refugee and migrant population on the Thai-Myanmar border. (DOCX) [file pone.0100247.s001.docx]

Supplementary Table S1: Birthweight (g) percentiles per completed gestational week for the refugee and migrant population on the Thai-Myanmar border

|  | 1 | 2.5 | 5 | 10 | 12.5 | 50 | 87.5 | 90 | 95 | 97.5 | 99 |
| --- | --- | --- | --- | --- | --- | --- | --- | --- | --- | --- | --- |
| 28 | 358.0 | 428.0 | 488.3 | 557.7 | 582.8 | 802.8 | 1022.7 | 1047.8 | 1117.3 | 1177.5 | 1247.6 |
| 29 | 546.9 | 622.5 | 687.5 | 762.5 | 789.5 | 1026.9 | 1264.2 | 1291.2 | 1366.2 | 1431.2 | 1506.8 |
| 30 | 734.3 | 815.5 | 885.2 | 965.7 | 994.7 | 1249.4 | 1504.1 | 1533.1 | 1613.6 | 1683.3 | 1764.5 |
| 31 | 919.0 | 1005.6 | 1080.2 | 1166.1 | 1197.1 | 1469.2 | 1741.2 | 1772.3 | 1858.2 | 1932.7 | 2019.4 |
| 32 | 1099.6 | 1191.8 | 1271.0 | 1362.5 | 1395.5 | 1684.9 | 1974.3 | 2007.3 | 2098.8 | 2178.0 | 2270.2 |
| 33 | 1274.8 | 1372.5 | 1456.6 | 1553.5 | 1588.5 | 1895.3 | 2202.1 | 2237.1 | 2334.0 | 2418.0 | 2515.7 |
| 34 | 1443.3 | 1546.6 | 1635.4 | 1737.8 | 1774.7 | 2098.9 | 2423.1 | 2460.1 | 2562.4 | 2651.2 | 2754.5 |
| 35 | 1603.7 | 1712.5 | 1806.1 | 1913.9 | 1952.9 | 2294.5 | 2636.0 | 2675.0 | 2782.8 | 2876.4 | 2985.2 |
| 36 | 1754.6 | 1868.9 | 1967.3 | 2080.6 | 2121.5 | 2480.5 | 2839.4 | 2880.3 | 2993.7 | 3092.0 | 3206.3 |
| 37 | 1894.5 | 2014.4 | 2117.4 | 2236.3 | 2279.2 | 2655.5 | 3031.8 | 3074.7 | 3193.5 | 3296.6 | 3416.5 |
| 38 | 2021.9 | 2147.3 | 2255.2 | 2379.5 | 2424.4 | 2818.0 | 3211.7 | 3256.6 | 3380.9 | 3488.8 | 3614.1 |
| 39 | 2135.4 | 2266.3 | 2378.9 | 2508.7 | 2555.6 | 2966.6 | 3377.6 | 3424.5 | 3554.3 | 3666.9 | 3797.8 |
| 40 | 2233.2 | 2369.7 | 2487.0 | 2622.3 | 2671.2 | 3099.6 | 3528.0 | 3576.9 | 3712.2 | 3829.5 | 3966.0 |
| 41 | 2314.0 | 2456.0 | 2578.1 | 2718.9 | 2769.7 | 3215.5 | 3661.3 | 3712.1 | 3852.9 | 3975.0 | 4117.0 |
